# Reassessing the association: Evaluation of a polyalanine deletion variant of *RUNX2* in non‐syndromic sagittal and metopic craniosynostosis

**DOI:** 10.1111/joa.14052

**Published:** 2024-05-17

**Authors:** Isaac S. Walton, Emma McCann, Astrid Weber, Jenny E. V. Morton, Peter Noons, Louise C. Wilson, Rosanna C. Ching, Deirdre Cilliers, David Johnson, Julie M. Phipps, Deborah J. Shears, Gregory P. L. Thomas, Steven A. Wall, Stephen R. F. Twigg, Andrew O. M. Wilkie

**Affiliations:** ^1^ MRC Weatherall Institute of Molecular Medicine John Radcliffe Hospital, University of Oxford Oxford UK; ^2^ Department of Clinical Genetics Liverpool Women's NHS Foundation Trust Liverpool, England UK; ^3^ West Midlands Regional Clinical Genetics Service and Birmingham Health Partners Birmingham Women's and Children's Hospitals NHS Foundation Trust Birmingham UK; ^4^ Birmingham Craniofacial Unit Birmingham Women's and Children's Hospitals NHS Foundation Trust Birmingham UK; ^5^ Clinical Genetics Service Great Ormond Street Hospital for Children NHS Foundation Trust London UK; ^6^ Oxford Craniofacial Unit Oxford University Hospitals NHS Foundation Trust Oxford UK; ^7^ Oxford Centre for Genomic Medicine Oxford University Hospitals NHS Foundation Trust Oxford UK; ^8^ Present address: Department of Clinical Genetics CHI at Crumlin Dublin Ireland

**Keywords:** craniosynostosis, genetic association, metopic synostosis, polyalanine, RUNX2, sagittal synostosis

## Abstract

The RUNT‐related transcription factor RUNX2 plays a critical role in osteoblast differentiation, and alterations to gene dosage cause distinct craniofacial anomalies. Uniquely amongst the RUNT‐related family, vertebrate *RUNX2* encodes a polyglutamine/polyalanine repeat (Gln_23_‐Glu‐Ala_17_ in humans), with the length of the polyalanine component completely conserved in great apes. Surprisingly, a frequent 6‐amino acid deletion polymorphism, p.(Ala84_Ala89)del, occurs in humans (termed 11A allele), and a previous association study (Cuellar et al. *Bone* **137**:115395;2020) reported that the 11A variant was significantly more frequent in non‐syndromic sagittal craniosynostosis (nsSag; allele frequency [AF] = 0.156; 95% confidence interval [CI] 0.126–0.189) compared to non‐syndromic metopic craniosynostosis (nsMet; AF = 0.068; 95% CI 0.045–0.098). However, the gnomAD v.2.1.1 control population used by Cuellar et al. did not display Hardy–Weinberg equilibrium, hampering interpretation. To re‐examine this association, we genotyped the *RUNX2* 11A polymorphism in 225 individuals with sporadic nsSag as parent–child trios and 164 singletons with sporadic nsMet, restricting our analysis to individuals of European ancestry. We compared observed allele frequencies to the non‐transmitted alleles in the parent–child trios, and to the genome sequencing data from gnomAD v.4, which display Hardy–Weinberg equilibrium. Observed AFs (and 95% CI) were 0.076 (0.053–0.104) in nsSag and 0.082 (0.055–0.118) in nsMet, compared with 0.062 (0.042–0.089) in non‐transmitted parental alleles and 0.065 (0.063–0.067) in gnomAD v.4.0.0 non‐Finnish European control genomes. In summary, we observed a non‐significant excess, compared to gnomAD data, of 11A alleles in both nsSag (relative risk 1.18, 95% CI 0.83–1.67) and nsMet (relative risk 1.29, 95% CI 0.87–1.92), but we did not replicate the much higher excess of *RUNX2* 11A alleles in nsSag previously reported (*p* = 0.0001).

## Graphical abstract

The skull images in the abstract require attribution as follows: “Skull image created by Servier Medical Art, and modified for use under a CC BY 4.0 license (https://creativecommons.org/licenses/by/4.0/)”.

The transcription factor RUNX2, a key regulator of osteogenic differentiation, contains a polyalanine repeat that is polymorphic in the human population. It was previously reported that individuals with non‐syndromic sagittal craniosynostosis are significantly enriched for the less frequent allele encoding 11 alanines. We reinvestigated this observation in an independent cohort of patients with either sagittal or metopic suture synostosis, but did not identify a significant association between alanine tract length and either phenotype.

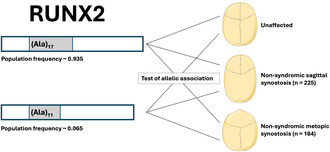

## 1 INTRODUCTION

Although great progress has been made in determining the genetic causes of syndromic craniosynostosis over the past 30 years, understanding the origins of non‐syndromic single suture craniosynostosis has lagged behind. The virtual absence of extensive pedigrees segregating these phenotypes demonstrates that these are rarely simple Mendelian conditions; instead, a complex mixture of environmental factors, polygenes, and monogenic alleles with reduced penetrance and variable expressivity are likely responsible, with different (but currently unmeasurable) relative contributions of these factors in different individuals who appear to be affected with similar phenotypes (Twigg & Wilkie, 2015).

Non‐syndromic craniosynostosis most commonly involves the midline sutures, with non‐syndromic metopic (nsMet) and sagittal (nsSag) synostosis accounting for 15%–20% and 35%–40% of all cases of craniosynostosis, respectively (Gaillard et al., 2023; Wilkie et al., 2017). Although the metopic and sagittal sutures themselves are both of neural crest origin, their bony margins have different origins; the frontal bones bordering the metopic suture are neural crest‐derived, whereas the parietal bones bordering the sagittal suture originate from cephalic mesoderm (Jiang et al., 2002). These differences in developmental embryology of the sutures could underlie differences in developmental pathology between nsMet and nsSag.

Although the genetic factors contributing to non‐syndromic midline craniosynostosis remain poorly characterised, a few robust observations have been made. In both nsMet and nsSag, heterozygous pathogenic variants in *SMAD6* are the most important monogenic (Mendelian) contributor, being observed in ~5% nsMet and ~1% nsSag (Calpena et al., 2020; Di Rocco et al., 2023; Timberlake et al., 2016, 2017). The role played by de novo mutations in other genes remains controversial (Kiziltug et al., 2023; Timberlake et al., 2017). Only two genome‐wide association studies (GWAS) of craniosynostosis have been published. In 2012, a GWAS of nsSag identified two significant loci, on chromosome 20 near *BMP2* and on chromosome 7 within *BBS9*, with the lead single‐nucleotide polymorphisms (SNPs) exhibiting highly significant odds ratios (4.38 and 4.17 in the meta‐analyses, respectively), compared to controls (Justice et al., 2012). A more recent similar study of nsMet identified one replicated genome‐wide significant SNP, located within *BMP7* (meta‐analysis odds ratio 1.74 compared to controls) (Justice et al., 2020).

The starting point for this work was a previous investigation of the *RUNX2* gene in nsSag and nsMet (Cuellar et al., 2020). *RUNX2*, one of three vertebrate family members related to the *Drosophila* gene *runt*, encodes a transcription factor that acts as a master regulator of osteoblast differentiation (Ducy et al., 1997; Otto et al., 1997). Haploinsufficiency of *RUNX2* causes cleidocranial dysplasia type 1 (CLCD1; OMIM 119600), a condition characterised by the reduced ossification of the skull and additional skeletal abnormalities including absent lateral portions of the clavicle, short stature, and severe dental anomalies (Lee et al., 1997; Mundlos et al., 1997). Contrastingly, increased *RUNX2* dosage is a known (but rare) cause of craniosynostosis (Greives et al., 2013; Mefford et al., 2010; Varvagiannis et al., 2013). Following exploratory sequencing of *RUNX2* in a small cohort of individuals with nsSag, Cuellar et al. (2020) undertook further targeted investigations of an 18‐nucleotide *RUNX2* deletion variant, NM_001024630.4:c.243_260del (rs11498192), encoding p.(Ala84_Ala89)del; this variant, subsequently referred to as 11A, lies within a long tract of imperfect triplet repeats that normally encode the sequence (Gln)_23_Glu(Ala)_17_, thus reducing the length of the polyalanine section from 17 (wild‐type [WT] allele), to 11 residues.

The results of the work by Cuellar et al. (2020), and their interpretation, are summarised in Table 1 (topmost three lines). A highly significant 2.3‐fold higher prevalence of 11A alleles was observed in nsSag (AF = 0.156) compared with nsMet (AF = 0.068) (*p* = 3.1 × 10^−4^). However, interpretation of this finding was impeded because the control population used as a comparator, from gnomAD v2.1.1 non‐Finnish European (NFE) individuals mostly analysed by exome sequencing (Table 1, third line), deviated grossly from Hardy–Weinberg equilibrium (HWE) and the gnomAD data were flagged as being suspect (Karczewski et al., 2020). The existence of a robust association between *RUNX2* 11A and the prevalence of nsSag would have major significance for understanding the pathogenesis of non‐syndromic craniosynostosis, so we decided to reinvestigate these observations, with the aim of first, establishing a reliable background allele frequency (AF) of 11A in the NFE population, and second, genotyping an independent cohort of nsSag and nsMet subjects, to see if we could replicate the original findings.

**TABLE 1:** Genotyping summary of *RUNX2* 11A deletion allele in Cuellar et al. (2020), gnomAD, and this work.

	Number of individuals	Number of alleles	Number of heterozygotes	Number of homozygotes	Hardy–Weinberg equilibrium test	Allele frequency	95% CI for allele frequency	Odds ratio (95% CI)^a^
nsSag (Cuellar et al., 2020)	270	540	84^b^		N/A	0.156	0.126‐0.189	2.65 (2.1–3.36)
nsMet (Cuellar et al., 2020)	191	382	26^b^		N/A	0.068	0.045‐0.098	1.05 (0.71–1.57)
gnomAD v2.1.1 (NFE)^c^	44,956	88,912	4973	1170	<1 e‐10	0.081	na	na
nsSag (this work)	225	450	32	1	0.79	0.076	0.053–0.104	1.18 (0.83–1.67)
nsSag – non‐transmitted allele from parent (this work)	450	450	28^b^		na	0.062	0.042‐0.089	0.96 (0.65–1.4)
nsMet (this work)	164	328	25	1	0.91	0.082	0.055–0.118	1.29 (0.87–1.92)
gnomAD v4.0.0 genomes (NFE)	33,852	67,704	4115	139	0.75	0.0649	0.063–0.067	na

Abbreviations: NFE, non‐Finnish European; N/A, not available; na, not applicable.

^a^ Odds ratios compared to gnomAD v4.0.0 genomes.

^b^ Total number of mutant alleles.

^c^ Data flagged in gnomAD v2.1.1 as likely inaccurate.

## 2 METHODS

### 2.1 Patients

Ethical approval was obtained from London‐Riverside Research Ethics Committee to collect peripheral blood samples for this work (reference 09/H0706/20). Written informed consent was provided to obtain all samples. DNA samples were either blood‐derived or extracted from Epstein–Barr transformed lymphocyte cultures. To match the cohorts used in Cuellar et al. (2020), 225 nsSag proband and parent trios were selected for the transmission disequilibrium test based on white ethnicity, unaffected parents, and no known genetic cause. The 164 nsMet singleton samples were selected solely on white ethnicity, and no known genetic cause.

### 2.2 Polymerase chain reaction (PCR)

Primers [forward (5′‐CCCTCCAGCAGCCTGCAGCCC‐3′) and reverse (5'‐GGTCGGCGATGATCTCCACCATGG‐3′)] were designed to GRCh38 chr6:45422622–45,422,859, yielding a 238 bp amplicon covering *RUNX2* exon 2, including the entire Gln_23_GluAla_17_ region. Roche FastStart™ Taq Polymerase was used with a modified master mix incorporating 20% dimethylsulphoxide, 1x FastStart™ Taq Reaction Buffer, 0.05 μM forward primer, 0.05 μM reverse primer, 0.5 mM dNTPs, 0.16 μL FastStart™ Taq DNA Polymerase, 20 ng DNA, and nuclease‐free water for a final reaction volume of 20 μL, amplified with optimised thermocycling (Supplementary Table S1). PCR products were separated by electrophoresis on a 4% agarose gel and scored for the deletion allele (Supplementary Figure S1A).

Samples with atypical results were re‐run and, if necessary, dideoxy‐sequenced following gel extraction (Monarch DNA Gel extraction kit). One nsMet proband screened was heterozygous for a 12 bp tandem duplication (c.174_185dup) in the polyglutamine‐encoding region upstream of 11A (Supplementary Figure S1B); sequencing of the polyalanine repeat confirmed a normal (WT/WT) genotype.

### 2.3 Statistical tests

A transmission disequilibrium test was applied to the nsSag trios (Spielman et al., 1993). Fisher's exact test was used when comparing allele frequencies. Confidence limits for allele frequencies were calculated following the binomial distribution. Confidence limits for odds ratios (Morris & Gardner, 1988) were calculated from the Select Statistical Services website (https://select‐statistics.co.uk/calculators/confidence‐interval‐calculator‐odds‐ratio/).

## 3 RESULTS

Initially, we genotyped the 11A deletion allele in 225 nsSag trios, each comprising a sporadically affected child and both their unaffected parents. Complete results are shown in Table 1 and Supplementary Figure S2. The observed AF in nsSag probands was 0.076, less than half the value obtained by Cuellar et al. (2020), a highly significant difference (*p* = 0.0001, Fisher's exact test). Fifty‐five parents were heterozygous for 11A and hence informative for transmission: 11A was transmitted in 31 instances and not transmitted in 24, yielding a transmission proportion of 0.56 (95% CI 0.42–0.7). The background 11A AF in the sampled population, estimated by counting the non‐transmitted alleles in these trios, was 0.062 (95% CI 0.042–0.089) (Table 1). This figure is very similar to the 11A AF in NFE gnomAD v.4.0.0 genomes (0.065; 95% CI 0.063–0.067); importantly unlike the equivalent exome data, the three WT/11A genotypes are present in HWE (Table 1). Using the gnomAD v.4.0.0 genome data as the control population, the prevalence of 11A alleles in nsSag was mildly but not significantly elevated (odds ratio 1.18, 95% CI 0.83–1.67).

Finally, we also examined the prevalence of 11A alleles in 164 subjects sporadically affected with nsMet (we did not genotype their parents). Similar to nsSag, we found a mildly but not significantly elevated odds ratio (1.29; 95% CI 0.87–1.92), compared to gnomAD v.4.0.0 genome controls (Table 1).

## 4 DISCUSSION

Given the role of *RUNX2* as a master regulator of osteogenesis and the usually high mutability of triplet repeats, the persistence of the poly(Gln_x_Ala_y_) motif in a wide range of vertebrate species is intriguing (Newton & Pask, 2020). Functional significance is also implied by the conservation of (Ala)_17_ copy number in all great apes, and the fact that a variety of different codons encode the Gln and Ala amino acids (thus helping to reduce, but not eliminate, the occurrence of replication slippage). Although transactivation studies have produced conflicting data, with inhibitory, neutral, or activating effects all reported with the 11A variant (Cuellar et al., 2020; Morrison et al., 2013; Thirunavukkarasu et al., 1998), overall these studies suggest a functional consequence of altering the length of the normal poly(Ala)_17_ motif, in part through altering binding to the partner protein Core binding factor beta (CBFB) (Morrison et al., 2013; Thirunavukkarasu et al., 1998). This naturally raises the question of whether the 11A variant might be associated with variation either in continuous phenotypic traits, or in disease states.

When considering this question, it is important to note that the 11A variant arises from deletion of one of a tandemly repeated pair of the 18‐nucleotide sequence G(GCG)(GCT)(GCG)_3_GC (where brackets enclose sequential alanine codons). Hence, it is likely that the 11A deletion has arisen independently multiple times during human evolution through replication slippage and therefore will not be tagged by linkage disequilibrium with a neighbouring SNP. The consequence is that any phenotypic effects of the 11A are unlikely to be detectable using conventional GWAS; rather, directed genotyping of the variant must be undertaken, either using a targeted assay (as presented here, see Supplementary Figure S1A) or by dideoxy‐ or next‐generation sequencing (NGS). Importantly, all three approaches may be associated with technical problems leading to inaccurate genotyping. Our PCR‐based gel electrophoresis method will also detect other rearrangements of the amplicon, which are predicted from gnomAD v.4.0.0 data collectively to occur at 7.4% of the frequency of 11A alone in the NFE population (Supplementary Table S2); indeed, we identified one atypical sequence based on aberrant migration of fragments on the gel (Supplementary Figure S1B). However, sequencing approaches (whether dideoxy‐ or NGS‐based) may be associated with systematic alignment and genotyping errors; undercalling of heterozygotes in low‐complexity regions is a known problem with NGS technology (Fuentes Fajardo et al., 2012). This is clearly illustrated by the gross deviation from HWE of the gnomAD v2.1.1 exome sequencing data used as a control by Cuellar et al. (2020). Interestingly, in the current version (v.4.1.0) of gnomAD, the 11A genotypes based on exome (but not genome) sequence continue to deviate grossly from HWE; this is accompanied by a “Discrepant frequencies” warning flag.

Notwithstanding these caveats, previous lines of evidence support that the 11A allele is associated with variation in several phenotypic traits. Morrison et al. (2013) reported that the 11A allele is associated with reduced serum collagen crosslinks, consistent with decreased bone turnover, and that 11A carriers were nearly twice as likely to have sustained fracture, with fractures biased towards bones of intramembranous rather than endochondral origin. Consistent with this, phenotype‐wide association studies (PheWAS) from UK Biobank data (Karczewski et al., 2022) identified significant associations of 11A with several bone‐related traits including heel bone mineral density, phosphate, standing and sitting height, and forced vital capacity (Supplementary Figure S3).

In the context of these observations, the proposed strong association of 11A with nsSag identified by Cuellar et al. (2020) is intriguing. However, we have not been able to replicate their observations in our own cohort, and we note several issues with the data reported by Cuellar et al. (2020), including inconsistencies in presentation of the numbers of genotypes obtained, variation in the genotyping method used for different cohorts, and failure to confirm HWE in either case or control data. Thus, we conclude that much larger studies will be required to confirm whether the *RUNX2* 11A variant is indeed associated with susceptibility to non‐syndromic craniosynostosis, and if so to confirm the effect size.

## AUTHOR CONTRIBUTIONS

Study conception and design: AOMW. Providing patient samples: EM, AW, JEVM, PN, LCW, RCC, DC, DJ, JMP, DJS, GPLT, SAW, AOMW. Experimental data collection: ISW. Analysis and interpretation: ISW, SRFT, AOMW. Draft manuscript preparation: ISW, AOMW. Critical review of manuscript: ISW, SRFT, AOMW.

## Supporting information


Data S1:

## Data Availability

The nsSag trio genotyping results that support the findings of this study are available in the supplementary material of this article. The raw data (4% agarose gel images and 11A allele scoring) for genotyping nsSag trios and nsMet samples that support the findings of this study are available from the corresponding author upon request.
