# Supplementary material for: Reassessing the association: Evaluation of a polyalanine deletion variant of RUNX2 in non‐syndromic sagittal and metopic craniosynostosis
Source: J Anat. 2024 May 17;245(6):874–8. doi: 10.1111/joa.14052 (PMC11547237; doi:10.1111/joa.14052)
Supplement: Supplementary file 1 — Data S1: [file JOA-245-874-s001.zip › joa14052-sup-0001-Supinfo/joa14052-sup-0001-Supinfo.docx]

**Reassessing the association: evaluation of a polyalanine deletion variant of *RUNX2* in non-syndromic sagittal and metopic craniosynostosis**, by Isaac S. Walton *et al*.

**Supplementary Material**

**
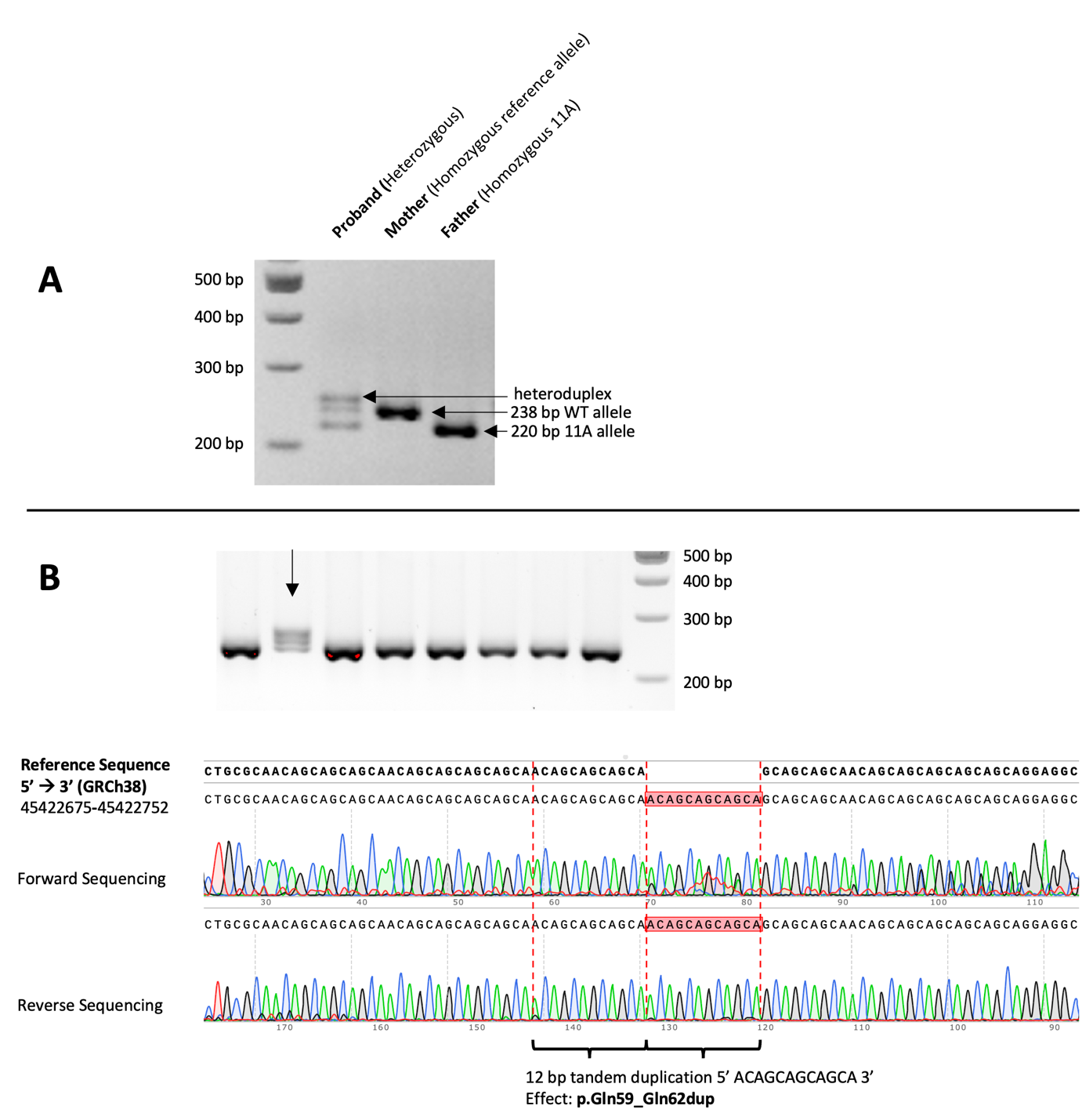
**

**Supplementary Figure 1. Genotyping of the 11A polymorphism by PCR and gel electrophoresis. A:** The pictured 4% gel shows an example where all 3 genotypes are visualised from family members of a single trio, with the 18 bp difference of 11A visible between samples. **B:** Atypical result from nsMet proband visualised on a 4% gel (upper panel). The central (insertion) band from this proband was gel extracted and Sanger-sequenced in both directions (lower panel), showing a 12 bp tandem duplication (c.174_185dup) in the polyglutamine-encoding region upstream of 11A.

**
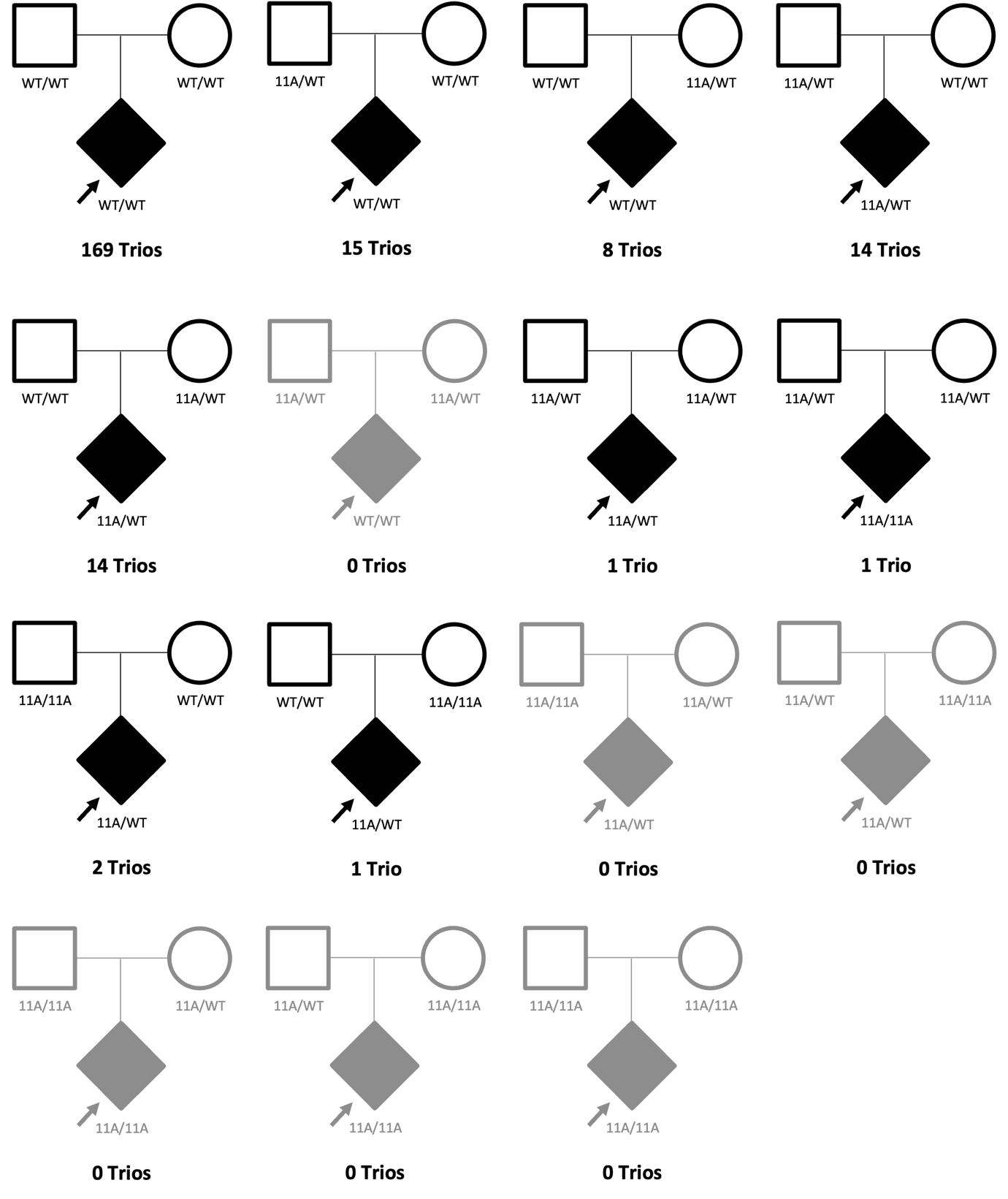
**

**Supplementary Figure 2. *RUNX2* 11A genotyping of 225 sporadically affected nsSag trios.** Unobserved pedigrees are depicted as greyed-out.

**
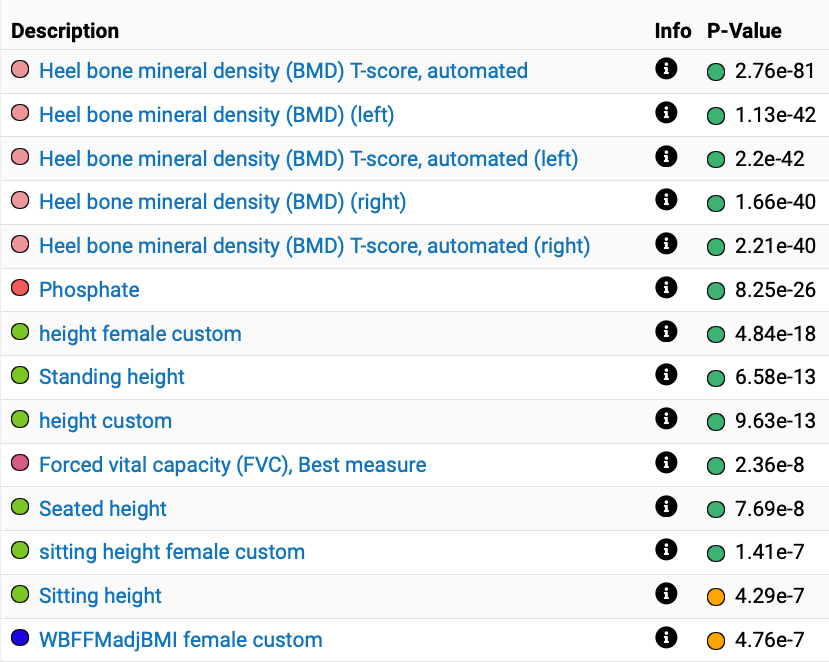
**

**Supplementary Figure 3. Associations with 11A genotype identified in UK Biobank PheWAS.** Screenshot taken on 05/02/2024 09:17 from Genebass v.0.13.0-bc4385f8-202303231340 (<https://app.genebass.org/gene/ENSG00000124813/phenotype/continuous-BMDadjBMI_custom-both_sexes--custom/variant/6-45422749-AGGCGGCGGCGGCGGCTGC-A?burdenSet=missense%7CLC&phewasOpts=1&resultIndex=variant-phewas&resultLayout=small>) showing all significant associations between 11A genotype and phenotypic traits measured in UK BioBank (Karczewski et al., 2022). *P*-Values were calculated using SKAT-O (Lee et al., 2012). ‘BMDasjBMI custom’, ‘bone mineral density custom’, and ‘BMDadjHtWt custom’ refer to the construction of a value for bone mineral density using the methodology of Kemp et al. (2017). ‘WBFFMadjBMI female custom’ refers to a body composition estimation of total fat-free mass in females, estimated by impedance measurement.

**Supplementary Table 1. Optimised thermocycling for genotyping *RUNX2* 11A using Roche FastStart™ Taq.**

| **Temperature (°C)** | **Time (minutes:seconds)** | **Notes** |
| --- | --- | --- |
| **95** | **5:00** |  |
| **95** | **0:30** | **x 14 cycles** |
| **60.5** | **0:30** |  |
| **72** | **0:45** |  |
| **95** | **0:30** | **x 25 cycles** |
| **60.5** | **0:30** |  |
| **72** | **0:45 (+ 5 second cumulative extension each cycle)** |  |
| **72** | **5:00** |  |
| **4** | **∞** |  |

**Supplementary Table 2. Copy number variation of *RUNX2* Gln_23_GluAla_17_ region included in the genotyping PCR for the 11A allele (yellow highlight), extracted from gnomAD v.4.0.0 genomes dataset.**

## Submitted as a separate Excel document.

## **Supplementary References**

KARCZEWSKI, K. J., SOLOMONSON, M., CHAO, K. R., GOODRICH, J. K., TIAO, G., LU, W., RILEY-GILLIS, B. M., TSAI, E. A., KIM, H. I., ZHENG, X., RAHIMOV, F., ESMAEELI, S., GRUNDSTAD, A. J., REPPELL, M., WARING, J., JACOB, H., SEXTON, D., BRONSON, P. G., CHEN, X., HU, X., GOLDSTEIN, J. I., KING, D., VITTAL, C., POTERBA, T., PALMER, D. S., CHURCHHOUSE, C., HOWRIGAN, D. P., ZHOU, W., WATTS, N. A., NGUYEN, K., NGUYEN, H., MASON, C., FARNHAM, C., TOLONEN, C., GAUTHIER, L. D., GUPTA, N., MACARTHUR, D. G., REHM, H. L., SEED, C., PHILIPPAKIS, A. A., DALY, M. J., DAVIS, J. W., RUNZ, H., MILLER, M. R. & NEALE, B. M. 2022. Systematic single-variant and gene-based association testing of thousands of phenotypes in 394,841 UK Biobank exomes. *Cell Genomics,* 2**,** 100168.

KEMP, J. P., MORRIS, J. A., MEDINA-GOMEZ, C., FORGETTA, V., WARRINGTON, N. M., YOULTEN, S. E., ZHENG, J., GREGSON, C. L., GRUNDBERG, E., TRAJANOSKA, K., LOGAN, J. G., POLLARD, A. S., SPARKES, P. C., GHIRARDELLO, E. J., ALLEN, R., LEITCH, V. D., BUTTERFIELD, N. C., KOMLA-EBRI, D., ADOUM, A.-T., CURRY, K. F., WHITE, J. K., KUSSY, F., GREENLAW, K. M., XU, C., HARVEY, N. C., COOPER, C., ADAMS, D. J., GREENWOOD, C. M. T., MAURANO, M. T., KAPTOGE, S., RIVADENEIRA, F., TOBIAS, J. H., CROUCHER, P. I., ACKERT-BICKNELL, C. L., BASSETT, J. H. D., WILLIAMS, G. R., RICHARDS, J. B. & EVANS, D. M. 2017. Identification of 153 new loci associated with heel bone mineral density and functional involvement of GPC6 in osteoporosis. *Nature Genetics,* 49**,** 1468-1475.

LEE, S., EMOND, M. J., BAMSHAD, M. J., BARNES, K. C., RIEDER, M. J., NICKERSON, D. A., DAVID, MARK & LIN, X. 2012. Optimal Unified Approach for Rare-Variant Association Testing with Application to Small-Sample Case-Control Whole-Exome Sequencing Studies. *The American Journal of Human Genetics,* 91**,** 224-237.
